# Effects of Exposure to Non-Immersive Virtual Reality on Disgust and Anxiety: A Study on Non-Clinical Samples

**DOI:** 10.3390/bs16030445

**Published:** 2026-03-18

**Authors:** Stefania Mancone, Francesco Di Siena, Simone Barbato, Lorenzo Di Natale, Fernando Bellizzi, Pio Alfredo Di Tore, Pierluigi Diotaiuti

**Affiliations:** 1 Department of Human Sciences, Society and Health, University of Cassino and Southern Lazio, 03043 Cassino, Italy; francesco.disiena@unicas.it (F.D.S.); fernando.bellizzi@unicas.it (F.B.); pioalfredo.ditore@unicas.it (P.A.D.T.); p.diotaiuti@unicas.it (P.D.); 2 Virtual Lab, IDEGO Digital Psychology Society, 00185 Rome, Italy; barbato@idego.it (S.B.); dinatale@idego.it (L.D.N.)

**Keywords:** screen-based virtual reality, non-immersive VR, emotion elicitation, disgust sensitivity, state/trait anxiety, obsessive–compulsive symptoms, contamination fear, COVID-19-related anxiety

## Abstract

Non-immersive (screen-based) virtual environments may offer a low-threshold way to elicit clinically relevant emotions while reducing barriers associated with immersive head-mounted displays. This study examined whether a non-immersive virtual scenario simulating a dirty public bathroom is associated with changes in disgust-related and anxiety-related responses in a non-clinical sample of university students, and explored links with obsessive–compulsive tendencies and contamination-related anxiety during the COVID-19 period. A total of 122 participants remotely explored the virtual environment. Before and after exposure, participants completed measures of state anxiety (EMAS-S) and disgust sensitivity (DSR); trait anxiety (EMAS-T), obsessive–compulsive symptoms (OCI-R), fear of COVID-19 (FCS), and engagement/activation during the experience were also assessed. Pre–post differences were tested using paired-sample *t*-tests, and associations among variables were examined via bivariate correlations. Results indicated a significant post-exposure increase in EMAS-S and DSR scores. Correlational analyses showed robust associations between disgust sensitivity and obsessive–compulsive symptoms, and between trait anxiety and fear of COVID-19. Gender and first-person videogame experience were related to subjective discomfort and activation, with higher levels reported by females and participants without gaming experience. The findings provide preliminary evidence that a remote, screen-based contamination scenario can elicit measurable disgust- and anxiety-related responses and that individual differences may shape subjective impact. However, because the study used a single-group pre–post design without a neutral VR condition or a non-VR control, causal conclusions about the effects of the virtual scenario cannot be drawn.

## 1. Introduction

Virtual reality (VR) has revolutionized numerous fields, from gaming to education, and is also emerging as a powerful tool in clinical psychology and rehabilitation. Several studies have demonstrated that VR can be effectively used for the rehabilitation of functional and cognitive abilities, offering patients a controlled and customizable environment in which to practice and improve specific skills (Moreno et al., 2019; Park et al., 2019; Voinescu et al., 2021; Zhu et al., 2021; Groenveld et al., 2022; De Luca et al., 2023; Ren et al., 2024; Moulaei et al., 2024). However, despite the promising results, the application of VR in complex clinical contexts still raises concerns, particularly regarding its acceptability among vulnerable populations such as the elderly or individuals with disabilities, who may find immersive systems too invasive or difficult to use (Dermody et al., 2020; Sagnier et al., 2020; Qi, 2024; Twamley et al., 2024).

Virtual reality (VR) offers vast potential in the field of clinical psychology, with applications ranging from therapeutic exposure to the treatment of anxiety disorders. However, it is important to distinguish between two main types of VR: immersive and non-immersive. Immersive VR, which uses headsets and goggles to create a fully enveloping experience, is particularly effective in eliciting intense emotional responses and replicating real-life conditions in therapeutic and training contexts. Nevertheless, this type of VR can be expensive, technologically complex, and potentially overwhelming for some users, especially those with sensory sensitivities or limited technological skills (Syed-Abdul et al., 2019; Dilanchian et al., 2021; Creed et al., 2024). On the other hand, non-immersive VR, which utilizes traditional screens and common input devices like keyboards and mice, offers a less immersive but more accessible and manageable experience. This type of VR, while not creating the same intensity of presence as immersive VR, allows for greater control and ease of use, making it ideal for a wider range of populations, including the elderly or those who might find immersive technology too invasive (Pallavicini et al., 2019; Wang et al., 2024; Ortiz-Mallasén, 2024). Besides, non-immersive VR is generally more cost-effective and requires fewer technological resources, making it a feasible solution for institutions with limited budgets or for applications requiring extended or repeated use (Rendevski et al., 2022; Gómez Bergin & Craven, 2023). In response to these challenges, non-immersive virtual reality emerges as an accessible and versatile solution. This approach eliminates the need for headsets or goggles, replacing them with a traditional monitor through which the user interacts with three-dimensional environments via simple input devices like a joystick. This mode reduces potential barriers to use while maintaining the ability to create engaging and stimulating experiences that can be leveraged for therapeutic purposes (Lehmann et al., 2020; Garay-Sanchez et al., 2021).

Among the emotions that can be elicited through VR, disgust holds particular interest due to its unique psychological and physiological responses. Disgust is a fundamental emotion from an evolutionary standpoint, being linked to the defense against the ingestion of potentially dangerous or contaminating substances (Oaten et al., 2009). It has been widely documented that disgust is not only a momentary reaction to specific stimuli but can also reflect a stable predisposition, influencing how individuals react in various contexts (Cisler et al., 2009; Russell & Giner-Sorolla, 2013; Olatunji et al., 2017; Liuzza et al., 2019; Peléšková et al., 2024; Söylemez & Kapucu, 2024). A high propensity for disgust has been associated with a range of psychopathological disorders, including anxiety disorders (such as specific phobias), eating disorders (such as bulimia and anorexia nervosa), sexual dysfunctions, and hypochondria (Anderson et al., 2021; Borg & de Jong, 2021; Santarelli et al., 2021; Winder et al., 2021; Bektas et al., 2022; Kiss et al., 2022; Brassard et al., 2023; Phillips et al., 2023; Hain & Stevenson, 2025; Mitchell et al., 2024). Particularly relevant is the connection between disgust and obsessive–compulsive disorder (OCD), where the avoidance of perceived contaminating stimuli can become a dominant and dysfunctional behavior (Knowles et al., 2018; Inozu et al., 2023; Millar et al., 2023; Wang et al., 2024).

Disgust sensitivity/proneness is typically conceptualized as a relatively stable individual difference, and the Disgust Scale–Revised (DS-R/DSR) is widely used as a trait-like index of disgust propensity across domains (Olatunji et al., 2007a; van Overveld et al., 2011). However, disgust responses also have an important state component and can vary as a function of situational salience and recent exposure to contamination cues (Olatunji et al., 2025). Accordingly, when administered immediately after a salient contamination-related experience, changes in DSR scores should be interpreted cautiously as reflecting short-term shifts in self-reported disgust proneness/accessibility rather than a durable change in an underlying trait (van Overveld et al., 2011; Olatunji et al., 2025).

Disgust sensitivity shows a robust and highly replicated sex difference, with women typically reporting higher levels of disgust than men across self-report measures and elicitor domains (Druschel & Sherman, 1999; Al-Shawaf et al., 2018). In addition, familiarity with interactive 3D environments may influence how strongly virtual stimuli are appraised and experienced. Research in VR has shown that prior videogame experience is associated with differences in subjective comfort and adaptation to virtual environments, including lower cybersickness and altered presence-cybersickness dynamics, suggesting that experience with visually dynamic, interactive media can change how virtual content is processed and tolerated (Weech et al., 2019; Weech et al., 2020; Hasibuan et al., 2025). Although much of this evidence comes from immersive VR, it supports the more general expectation that users’ prior experience with virtual environments may influence engagement, perceived realism, and emotional impact, even in screen-based paradigms. Accordingly, beyond examining pre-post changes in anxiety and disgust responses, we also explored whether gender and first-person videogame experience were associated with subjective discomfort/activation during the non-immersive virtual exposure.

Although VR-based procedures have been widely used to elicit and modulate emotional responses in mental-health–relevant contexts (Emmelkamp & Meyerbröker, 2021; Gutiérrez-Maldonado, 2022; Bell et al., 2024), evidence also indicates that contamination-related virtual exposure can reliably activate disgust/anxiety and related urges in both healthy and clinical/non-clinical samples (Inozu et al., 2020; Ferraioli et al., 2024). Importantly, similar affective activation is not exclusive to fully immersive head-mounted displays, as non-immersive virtual environments can elicit measurable stress-related responses as well (Alghamdi et al., 2017). Therefore, our study does not claim novelty on the basis that VR can elicit disgust or anxiety per se; rather, it addresses a more specific and translationally relevant question: what is the added value and what are the boundary conditions of a remote, screen-based non-immersive paradigm for eliciting contamination-related affect.

Focusing on a non-immersive implementation is not merely a technical simplification, but a theoretically and practically meaningful choice. Compared to immersive setups, screen-based delivery may reduce barriers related to usability, accessibility, and adverse effects (e.g., cybersickness), potentially increasing feasibility and scalability across settings and user groups (Creed et al., 2024; Dilanchian et al., 2021) and aligning with cost and resource constraints often discussed in applied XR deployments (Gómez Bergin & Craven, 2023). Evidence from other clinical domains suggests that immersive and non-immersive formats may yield different profiles of effects, highlighting the importance of empirically testing non-immersive paradigms rather than generalizing from immersive VR findings (Garay-Sanchez et al., 2021).

In this framework, the present study evaluates a remote, screen-based contamination scenario with a controlled increase in visible dirtiness and repeated discomfort ratings, and examines whether responses within this paradigm map onto relevant vulnerability dimensions and individual moderators. In the present study, we use the term immedesimation (from the Italian “immedesimazione”) to refer to a process of situational self-identification, namely the extent to which participants adopt a first-person perspective and mentally place themselves in the depicted scenario. This construct is related to, but distinct from, immersion. In the virtual reality literature, immersion generally refers to the objective features of a system that determine how inclusive, vivid, and surrounding an environment is, whereas self-identification with a scenario concerns the user’s subjective perspective-taking and experiential involvement. Thus, in the present non-immersive, screen-based setting, the construct of interest is not immersion in the technological sense, but rather participants’ tendency to place themselves in the virtual situation and experience it from a first-person standpoint (Slater & Wilbur, 1997; Kilteni et al., 2012; Cummings & Bailenson, 2016).

In light of these premises, the present study aims to explore the emotional activation of disgust through a non-immersive virtual environment, using a scenario specifically designed to evoke this emotion in a sample of university students. The choice of a non-clinical sample is motivated by the intention to understand how individuals without psychopathological diagnoses respond to such stimuli, laying the groundwork for potential future clinical applications. The pandemic context in which the study was conducted, characterized by heightened sensitivity to contamination and hygiene issues due to COVID-19, offers a unique opportunity to examine how disease-related fears interact with the propensity for disgust and obsessive-compulsive behaviors (Miłkowska et al., 2021; Paluszek et al., 2021; Stevenson et al., 2021).

Accordingly, the present study aims to test whether a remote, screen-based non-immersive virtual exposure can elicit measurable changes in DSR scores (self-reported disgust proneness) and state anxiety, and to clarify the added value of this specific implementation by examining (1) associations between emotional activation and vulnerability dimensions relevant to contamination-related symptomatology (e.g., obsessive–compulsive tendencies and contamination-related fear) and (2) potential moderators of response with particular relevance for screen-based delivery (e.g., gender and familiarity with first-person videogames).

## 2. Materials and Methods

### 2.1. Participants

Participants for this study were selected through a voluntary recruitment process targeting university students. The selection criteria included the following: participants had to be between the ages of 18 and 38, without any ongoing psychopathological diagnoses. In addition, participants needed to demonstrate a willingness to participate in online experimental sessions. The final sample consisted of 122 individuals, evenly divided by gender (61 males and 61 females), with an average age of 23.4 years (SD = 6.10). An additional criterion was considered for half of the participants (51.6%), who reported having prior experience with first-person videogames. This criterion was included to analyze potential individual differences in reactions to the virtual environment. The students were reached through university channels, including email distributions, online student forums, and social media platforms associated with the university. Invitations to participate were sent out, highlighting the study’s objectives and requirements. Interested students were directed to an online registration platform where they could express their willingness to participate and provide preliminary consent. This approach ensured a broad reach within the university population, allowing for a diverse sample of students to be included in the study.

### 2.2. Procedure

**Figure 1:**
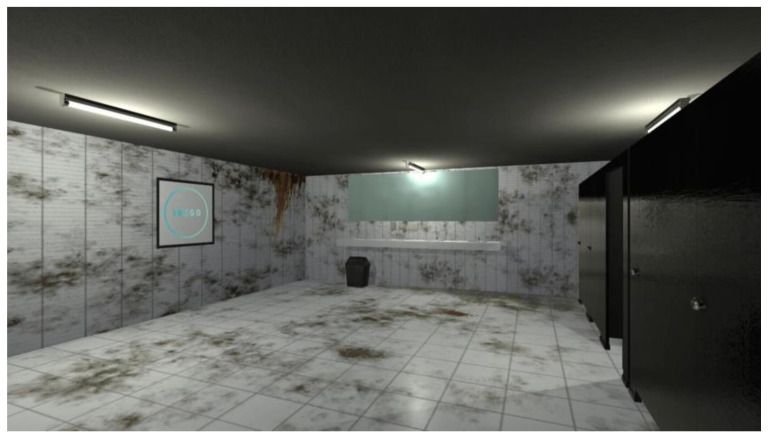
Visual representation of the virtual bathroom scenario used in the study, characterized by degraded hygienic conditions, including dirty floors, walls, and fixtures. This environment was designed to elicit strong emotional responses, particularly disgust and anxiety, among participants.

Participants took part in the study remotely, connecting to the DOCURE IDEGO online platform (https://www.idego.it/portfolio-item/docure/ accessed on 12 December 2020), which offers virtual simulations organized into two main categories of stimuli: disorder and dirt. For this research, the “Bathroom” scenario was selected, which simulates a public bathroom characterized by degraded hygienic conditions (see Figure 1).

The experimental procedure was divided into the following phases:

Briefing and Informed Consent: before starting the experiment, participants were provided with a detailed explanation of the study’s objectives and procedures, followed by the collection of written informed consent.

Pre-Test: participants completed a series of online questionnaires using the Questbase platform, including: (1) Endler Multidimensional Anxiety Scales-State (EMAS-S) to measure state anxiety; (2) Disgust Scale Revised (DSR) to assess the predisposition to disgust. (3) A preliminary assessment of the discomfort perceived when having to use a public bathroom (scale 0–100). This baseline item was intended to capture participants’ general anticipated discomfort about using a public bathroom and is conceptually distinct from the in-scenario discomfort indices collected during virtual exposure.

Exploration of the Virtual Environment: Participants were guided by an operator through a virtual scenario that recreated a dirty public bathroom. The experiential path included the following steps: (1) Entry into the anteroom: introduction to the virtual environment; (2) Access to the toilet stall: participants were asked to imagine having to use the bathroom; (3) Activation of the dirt level: through specific buttons, participants increased the visible dirt level in the toilet and sink. (4) Discomfort Assessment: during the exploration, participants provided brief real-time discomfort ratings on a 0 (“no discomfort”) to 100 (“extreme discomfort”) scale. Ratings were collected at predefined moments that correspond to the indices reported in the Results: (a) General Bathroom Discomfort (GBD) was assessed upon first viewing the bathroom environment; (b) Immedesimation/Identification-related Discomfort (ID) was assessed immediately after participants were instructed to imagine themselves in the depicted situation, namely having to use the bathroom in the virtual public toilet. Here, immedesimation was operationalized as situational self-identification with the scenario rather than immersion in the technological sense. Participants were therefore asked to adopt a first-person perspective and rate the discomfort elicited by this self-referential involvement on a 0–100 scale; (c) Dirty Toilet Bowl Discomfort (DTBD) was assessed after participants increased the visible dirt level in the toilet area, using two toilet-related ratings that were averaged to obtain DTBD; and (d) Dirty Sink Discomfort (DSD) was assessed after participants increased the visible dirt level in the sink area, using three sink-related ratings that were averaged to obtain DSD. All ratings were prompted by the operator and recorded in real time via a dedicated online form. These assessments were collected in real time by the operator through a specific form.

Post-Test: after the virtual experience, participants completed the EMAS-S and DSR again for a pre-post comparison and completed additional questionnaires: (1) Endler Multidimensional Anxiety Scales-Trait (EMAS-T) to assess trait anxiety; (2) Obsessive-Compulsive Inventory (OCI-R) to measure the predisposition to obsessive-compulsive behaviors; (3) Fear of COVID-19 Scale (FCS) to assess COVID-19-related anxiety; (4) Activation and Engagement Index (AEI) to measure the level of immersion and engagement during the virtual experience. The study was conducted in accordance with the requirements of the Declaration of Helsinki and approved by the local Institutional Review Board of the University of Cassino and Southern Lazio (IRB_DIPSUS 11: 20 February 2020). Written informed consent was obtained from all participants.

### 2.3. Psychometric Instruments

Endler Multidimensional Anxiety Scales (EMAS, Endler et al., 1991; Italian valid. Comunian, 1991/1996) assesses different dimensions of anxiety, both as a transient state and as a stable trait. The scales have been widely used in research and clinical settings to measure anxiety in response to specific situations and general tendencies. The EMAS has been validated in various languages, including Italian. EMAS-S (State Anxiety): this subscale consists of 20 items and measures the transient emotional state of anxiety related to a specific situation. It includes subscales for autonomic anxiety (AE) and cognitive worry (CW). In this study, the pre-test reported a Cronbach’s alpha of 0.93, and the post-test a Cronbach’s alpha of 0.95, indicating excellent internal consistency. EMAS-T (Trait Anxiety): the trait anxiety subscale includes 60 items that are divided into four situational categories: Social Evaluation, Physical Danger, Ambiguous Situations, and Daily Routine. This scale has shown a Cronbach’s alpha of 0.93, demonstrating high reliability in assessing stable anxiety traits.

Disgust Scale Revised (DSR, Olatunji et al., 2007b; Italian valid. Melli et al., 2013), is a psychometric tool designed to measure individual differences in disgust sensitivity. The scale is composed of 25 items, rated on a 5-point Likert scale, and is divided into three subscales: Core Disgust that assesses the fundamental emotional response to potentially contaminating stimuli; Animal Reminder that measures disgust sensitivity related to reminders of animal origins and mortality; Contamination Disgust that evaluates the aversion to potential sources of contamination, such as dirty environments or spoiled food. In the present study, administering the DSR both pre- and post-exposure was intended to capture short-term modulation in self-reported disgust proneness following contamination-cue exposure, rather than to imply a stable change in trait disgust sensitivity (van Overveld et al., 2011; Olatunji et al., 2025). In this study, the DSR demonstrated good internal consistency, with a Cronbach’s alpha of 0.80 in the pre-test and 0.82 in the post-test.

Obsessive-Compulsive Inventory (OCI-R, Foa et al., 2002; Italian valid. Marchetti et al., 2010), is a widely used self-report measure designed to assess the severity and type of obsessive-compulsive symptoms across multiple dimensions. The OCI-R consists of 18 items rated on a 5-point Likert scale, and it includes subscales for the following dimensions: Checking: measures the compulsion to check things repeatedly; Washing: assesses compulsive washing behaviors; Ordering: evaluates the need to arrange things in a particular order; Hoarding: measures the tendency to collect and keep unnecessary items; Mental Neutralization: assesses the use of mental rituals to neutralize distressing thoughts; Obsessive Rumination: evaluates persistent and intrusive thoughts. In this study, the OCI-R demonstrated good internal consistency, with a Cronbach’s alpha of 0.87.

Fear of COVID-19 Scale (FCS, Ahorsu et al., 2020; Italian valid. Soraci et al., 2020), is a psychometric tool specifically designed to assess anxiety related to the COVID-19 pandemic. The scale consists of 7 items rated on a 5-point Likert scale, which measures the intensity of fear associated with the virus and its consequences. In this study, the FCS demonstrated excellent internal consistency, with a Cronbach’s alpha of 0.90, indicating a high level of reliability in measuring COVID-19-specific anxiety.

Activation and Engagement Index (AEI). The AEI is an author-developed, study-specific measure designed to assess participants’ perceived engagement and activation during the virtual experience. It consists of 8 items rated on a 5-point Likert scale assessing perceived immersion, interactivity, realism, and sensory involvement (visual, olfactory, gustatory, auditory, and tactile impressions). Higher scores indicate greater perceived activation and engagement with the virtual environment. The items were: (1) I felt immersed in the virtual bathroom scenario; (2) The experience captured my attention and kept me mentally engaged; (3) I had the impression of interacting with the virtual environment rather than simply observing it; (4) The virtual bathroom environment appeared realistic to me; (5) The visual details of the environment made the experience feel vivid; (6) I had the impression of perceiving sensory cues (e.g., smells or tastes) related to the situation; (7) The sounds associated with the scenario contributed to making the experience engaging; (8) I could almost feel the physical sensations associated with being in that environment. The overall AEI score was calculated as the mean of the eight items, with higher values indicating greater activation and engagement. Given its novel nature, the AEI is considered an exploratory measure in the present study and is interpreted cautiously. To explore its dimensionality, an exploratory factor analysis (EFA) was conducted in SPSS on the 8 items. Prior to extraction, the suitability of the correlation matrix for factor analysis was evaluated using standard factorability checks (e.g., Kaiser–Meyer–Olkin measure and Bartlett’s test of sphericity). The number of factors to retain was determined using conventional criteria (e.g., eigenvalues > 1 and inspection of the scree plot), and items were considered adequate indicators if they showed salient loadings (≥0.40). The EFA suggested a predominantly unidimensional structure, with item loadings ranging from 0.65 to 0.80. Internal consistency in the present sample was acceptable (Cronbach’s α = 0.77). As the AEI has not yet undergone full psychometric validation (e.g., confirmatory factor analysis in an independent sample; convergent/discriminant validity; test–retest reliability), its scores are used here for descriptive and exploratory purposes only, in line with scale-development recommendations (Boateng et al., 2018; Fabrigar et al., 1999).

### 2.4. Data Analysis

Statistical analyses were conducted using SPSS (Statistical Package for the Social Sciences) version 26. The techniques used include: descriptive Statistics (means, standard deviations, and frequencies) to describe the sample and the variables studied; Univariate ANOVA used to examine differences between groups based on demographic and psychometric variables, such as gender and videogame experience; Bivariate Pearson and Spearman Correlations used to explore the relationships between key variables in the study, such as perceived discomfort, state and trait anxiety, fear of COVID-19, and obsessive-compulsive tendencies. Correlations were considered significant at *p* < 0.05 and *p* < 0.01. Paired Sample *t*-tests were conducted to compare levels of state anxiety and disgust sensitivity before and after exposure to the virtual environment, verifying the statistical significance of the differences observed. Internal consistency was evaluated using Cronbach’s α for the main scales (pre and post where applicable). In addition, an exploratory factor analysis (EFA) was performed on the AEI items to examine its dimensionality. To reduce overreliance on statistical significance, correlational findings are reported and interpreted primarily in terms of effect size (Pearson’s r) and the overall pattern of associations. We note that statistically significant correlations may still be small in magnitude and should not be automatically interpreted as psychologically or clinically meaningful. All correlational analyses are therefore treated as exploratory, and no causal or mechanistic conclusions are drawn from these associations. This approach follows recommendations emphasizing estimation and effect sizes over dichotomous interpretations based on *p*-values alone (Wasserstein & Lazar, 2016; Cumming, 2014). To examine whether the magnitude of pre-post change in state anxiety (EMAS-S) and disgust sensitivity (DSR) was associated with bathroom-specific discomfort variables, we computed individual change scores (Δ = post − pre) and tested them in multiple regression models (ANCOVA-equivalent approach) in which GBD, ID, DTBD, and DSD were entered simultaneously, controlling for gender and first-person videogame experience. In addition to paired-sample comparisons, we conducted mixed repeated-measures ANOVAs with Time (pre vs. post) as the within-subject factor and Gender (women vs. men) and first-person videogame experience (yes vs. no) as between-subject factors. For each outcome (EMAS-S and DSR), we examined the main effect of Time and the Time × Gender, Time × Videogame experience, and Time × Gender × Videogame experience interactions to test whether the pre–post change differed across groups. Effect sizes are reported as Cohen’s d for paired-sample *t*-tests, partial eta squared (ηp^2^) for ANOVA effects, and R^2^ for regression models. To contextualize the interpretation of both significant and null findings, a sensitivity power analysis was conducted for the main statistical tests. Following standard recommendations for power analysis in behavioral research (Cohen, 1988; Faul et al., 2007), the analysis assumed a two-tailed α level of 0.05 and a desired statistical power of 0.80. Given the available sample size (N = 122), the minimum detectable effect size was approximately dz = 0.26 for paired-sample pre–post comparisons and r = 0.25 for bivariate correlations. For the multiple regression models predicting change scores from bathroom discomfort indices while controlling for gender and first-person videogame experience (six predictors; complete-case N = 117), the minimum detectable overall effect size was approximately f^2^ = 0.12, corresponding to a small-to-medium effect according to conventional benchmarks (Cohen, 1988). These estimates indicate that the study was adequately powered to detect small-to-moderate within-subject effects, whereas smaller correlational or multivariable effects may not have been reliably detectable.

## 3. Results

### 3.1. Bivariate Correlations

Because multiple correlations were examined, the results are summarized with a focus on effect-size magnitude rather than statistical significance alone. Several associations reached statistical significance but were small in magnitude, and should be interpreted as modest relationships that may be sensitive to sample characteristics and measurement noise. Accordingly, we highlight only the most consistent patterns and avoid overinterpretation of small effects, consistent with guidance on effect-size evaluation in psychological research (Funder & Ozer, 2019; Gignac & Szodorai, 2016). Table 1 reports bivariate associations among perceived discomfort in the virtual bathroom (GBD, ID, DTBD, DSD), state anxiety (EMAS-S), trait anxiety (EMAS-T), fear of COVID-19 (FCS), obsessive-compulsive symptoms (OCI-R), and disgust sensitivity (DSR). Correlation coefficients (Pearson’s r or Spearman’s ρ, depending on distributional assumptions) are shown in the lower diagonal, and the corresponding two-tailed *p*-values are reported in the upper diagonal.

General Bathroom Discomfort (GBD). GBD vs. ID, DTBD, DSD: the correlations be-tween general discomfort in the bathroom and the other measures of discomfort (imme-desimation, dirty toilet bowl, and dirty sink) are all positive and significant. This indicates that participants who experience a high level of general discomfort also tend to experience greater specific discomfort in the other conditions of the virtual bathroom. This suggests consistency in individuals’ emotional reactions to the dirty virtual environment.

Immedesimation Bathroom Discomfort (ID). ID vs. EMAS-T: the positive correlation between immedesimation discomfort and trait anxiety (0.195*) suggests that individuals with a stable predisposition to anxiety (trait anxiety) tend to experience greater discomfort when imagining themselves having to use a dirty bathroom, highlighting the sensitivity of these individuals to potentially stressful or disgusting situations.

ID vs. OCI-R. The positive correlation with obsessive-compulsive behaviors (0.189*) indicates that individuals with a greater tendency towards obsessive-compulsive behavior experience more discomfort in situations that require immedesimation, suggesting a link between the perception of contamination and the compulsive response.

Dirty Toilet Bowl Discomfort (DTBD). DTBD vs. FCS, OCI-R, DSR: the correlations with fear of COVID-19 (0.189**), obsessive-compulsive behaviors (0.243**), and disgust sensitivity (0.364**) indicate that exposure to a dirty toilet in the virtual context is particularly activating for individuals with a high fear of contamination and high sensitivity to disgust. This reflects how extreme dirtiness can evoke intense emotional responses in pre-disposed individuals.

Dirty Sink Discomfort (DSD). DSD vs. OCI-R, DSR: the significant correlations with obsessive-compulsive behaviors (0.207*) and disgust sensitivity (0.317**) confirm that the dirty sink in the virtual context is also perceived as highly disturbing, especially by individuals with a predisposition to disgust and obsessive-compulsive behaviors.

State Anxiety (EMAS-S). EMAS-S vs. FCS, DSR: the correlations with fear of COVID-19 (0.267**) and disgust sensitivity (0.192**) indicate that state anxiety is influenced by fear of contamination and a predisposition to disgust, highlighting how the pandemic situation has amplified anxious responses in contexts perceived as contaminated.

Trait Anxiety (EMAS-T). EMAS-T vs. FCS, OCI-R, DSR: the correlations with fear of COVID-19 (0.452**), obsessive-compulsive behaviors (0.206*), and disgust sensitivity (0.245**) suggest that trait anxiety, being a stable predisposition, is strongly associated with these other variables, indicating that individuals with high trait anxiety are more likely to perceive dirty virtual situations as threatening or disgusting.

Fear of COVID-19 Scale (FCS). FCS vs. OCI-R, DSR: the correlations with obsessive-compulsive behaviors (0.424**) and disgust sensitivity (0.331**) emphasize the inter-connection between fear of viral contamination and obsessive-compulsive responses, suggesting that the pandemic context has amplified these responses in predisposed individuals.

Obsessive-Compulsive Inventory (OCI-R). OCI-R vs. DSR: the positive correlation be-tween obsessive-compulsive behaviors and disgust sensitivity (0.277**) confirms that individuals with higher obsessive–compulsive tendencies are also more sensitive to disgust, suggesting a potential target for therapeutic interventions using VR.

### 3.2. Paired t-Test Results

**Figure 2:**
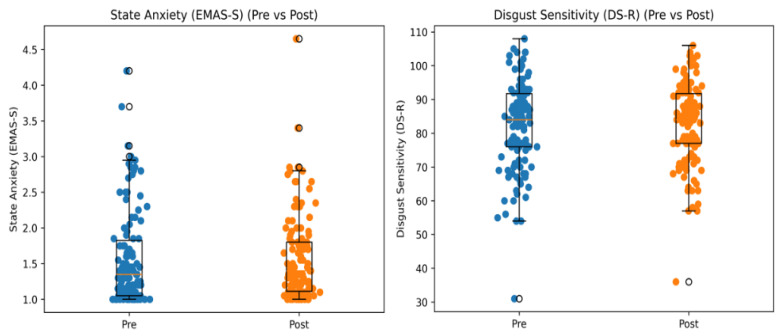
Distribution of pre- and post-exposure scores for state anxiety and disgust sensitivity. Individual observations are displayed as jittered points in the foreground, while boxplots in the background summarize the median and interquartile range.

The paired sample *t*-test revealed a significant increase in both state anxiety levels and disgust sensitivity after exposure to the virtual environment (see Figure 2).

State Anxiety (EMAS-S): M_pre = 2.43 (SD = 0.87), M_post = 3.30 (SD = 0.46), t(121) = −3.228, increase = 0.87, 95% CI [0.337, 1.403], *p* < 0.05, d = 0.54.

Disgust Sensitivity (DSR): M_pre = 2.24 (SD = 0.85), M_post = 3.17 (SD = 0.61), t(121) = −4.169, increase = 0.93, 95% CI [0.488, 1.372], *p* < 0.05, d = 0.61.

These results indicate that exposure to the dirty bathroom scenario was associated with a significant increase in both state anxiety and disgust sensitivity. However, given the absence of a neutral VR condition or a non-VR control group, the observed pre–post changes cannot be uniquely attributed to the virtual scenario.

#### 3.2.1. Moderation of Pre-Post Change in State Anxiety by Bathroom Discomfort Indices

To examine whether changes in state anxiety were more strongly related to bathroom-specific discomfort indices, we computed an individual change score for state anxiety (ΔEMAS-S = post − pre) and tested its association with General Bathroom Discomfort (GBD), Immedesimation/Identification-related Discomfort (ID), Dirty Toilet Bowl Discomfort (DTBD; mean of the two toilet-bowl items), and Dirty Sink Discomfort (DSD; mean of the three sink items). A multiple regression model (ANCOVA-equivalent with two repeated measurements) included all four discomfort indices simultaneously and controlled for gender and first-person videogame experience. The overall model was not significant, F(6, 110) = 0.46, *p* = 0.833, R^2^ = 0.025. None of the bathroom discomfort indices uniquely predicted the magnitude of pre–post change in state anxiety (GBD: β = −0.01, *p* = 0.920; ID: β = 0.09, *p* = 0.442; DTBD: β = −0.04, *p* = 0.781; DSD: β = −0.10, *p* = 0.502). Gender and videogame experience were also not associated with ΔEMAS-S (both *p* > 0.60). These analyses do not support the interpretation that the pre–post change in state anxiety is differentially explained by specific bathroom discomfort dimensions once shared variance among the discomfort indices and demographic covariates is taken into account.

#### 3.2.2. Moderation of Pre-Post Change in Disgust Sensitivity by Bathroom Discomfort Indices

To examine whether changes in disgust sensitivity were more strongly related to bathroom-specific discomfort indices, we computed an individual change score for disgust sensitivity (ΔDISGUST_DRS = post − pre) and tested its association with General Bathroom Discomfort (GBD), Immedesimation/Identification-related Discomfort (ID), Dirty Toilet Bowl Discomfort (DTBD; mean of the two toilet-bowl items), and Dirty Sink Discomfort (DSD; mean of the three sink items). A multiple regression model (ANCOVA-equivalent with two repeated measurements) included all four discomfort indices simultaneously and controlled for gender and first-person videogame experience (N = 117 with complete data). The overall model was significant, F(6, 110) = 3.00, *p* = 0.009, R^2^ = 0.140. Among the bathroom discomfort indices, only DSD uniquely predicted the magnitude of pre–post change in disgust sensitivity (β = −0.39, *p* = 0.007), indicating that higher sink-related discomfort was associated with a smaller increase (or a tendency toward a decrease) in DISGUST_DRS from pre to post exposure. The other indices did not show unique associations with ΔDISGUST_DRS (GBD: β = 0.03, *p* = 0.805; ID: β = −0.05, *p* = 0.651; DTBD: β = 0.12, *p* = 0.409). Gender showed a trend-level association (β = −0.18, *p* = 0.068), whereas videogame experience was not associated with ΔDISGUST_DRS (β = 0.03, *p* = 0.762). These results suggest that, when shared variance among bathroom discomfort indices and demographic covariates is accounted for, sink-related discomfort (DSD) is the only bathroom dimension that explains variability in the pre–post change in disgust sensitivity in this sample.

#### 3.2.3. Mixed Repeated-Measures ANOVA (Time × Gender × Videogame Experience)

To test whether pre–post changes following exposure were moderated by Gender and first-person videogame experience, we conducted mixed repeated-measures analyses with Time (pre vs. post) as the within-subject factor and Gender (women vs. men) and Videogame experience (yes vs. no) as between-subject factors (complete data: N = 117). Effect sizes are reported as partial eta squared (ηp^2^).

For state anxiety (EMAS-S), the main effect of Time was not significant, F(1, 116) = 0.00, *p* = 0.946, ηp^2^ < 0.001, indicating no reliable overall pre–post change in this sample. In addition, there was no evidence that change over time differed by Gender (Time × Gender: F(1, 113) = 0.22, *p* = 0.642, ηp^2^ = 0.002) or by Videogame experience (Time × Videogame: F(1, 113) = 0.53, *p* = 0.469, ηp^2^ = 0.005), and the three-way interaction was also not significant (Time × Gender × Videogame: F(1, 113) = 0.15, *p* = 0.698, ηp^2^ = 0.001).

For disgust sensitivity (DISGUST_DRS), there was a significant main effect of Time, F(1, 116) = 40.76, *p* < 0.001, ηp^2^ = 0.260, reflecting higher post-exposure scores compared to baseline. Importantly, the Time × Gender interaction was significant, F(1, 113) = 4.46, *p* = 0.037, ηp^2^ = 0.038, indicating that the pre–post increase in DISGUST_DRS was larger for men (ΔM ≈ 0.90) than for women (ΔM ≈ 0.41). The Time × Videogame experience interaction was not significant, F(1, 113) = 0.13, *p* = 0.720, ηp^2^ = 0.001, and the three-way Time × Gender × Videogame experience interaction was also not significant, F(1, 113) = 0.97, *p* = 0.326, ηp^2^ = 0.009.

### 3.3. Gender and Videogame Experience

**Figure 3:**
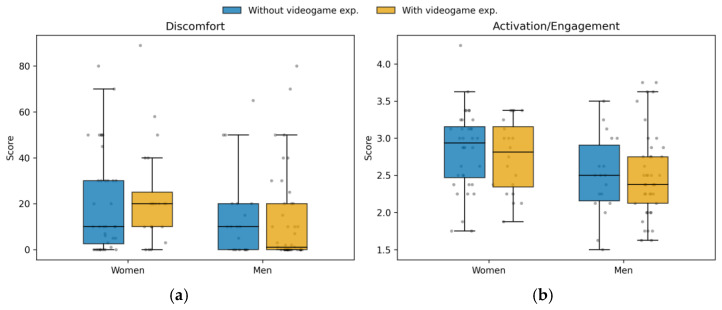
(**a**,**b**): Boxplots presented in two panels (Discomfort and Activation) reflecting the 2 × 2 design (Gender × Videogame Experience).

To test the 2 × 2 design, we conducted two-way ANOVAs with Gender (women vs. men) and first-person videogame experience (yes vs. no) as between-subject factors, separately for Discomfort (0–100 perceived discomfort rating) and Activation/Engagement (AEI score) (complete data: N = 117). For Discomfort, the Gender × Videogame experience interaction was not significant, F(1, 113) = 0.40, *p* = 0.530, ηp^2^ = 0.003, and the main effect of videogame experience was not significant, F(1, 113) = 0.01, *p* = 0.906, ηp^2^ < 0.001. The main effect of gender showed a small trend, with women reporting higher discomfort than men, F(1, 113) = 3.56, *p* = 0.062, ηp^2^ = 0.031 (women: M = 21.64; men: M = 13.72). For Activation/Engagement, the Gender × Videogame experience interaction was not significant, F(1, 113) = 0.21, *p* = 0.651, ηp^2^ = 0.002, and the main effect of videogame experience was not significant, F(1, 113) = 0.15, *p* = 0.696, ηp^2^ = 0.001. In contrast, the main effect of gender was significant, with women reporting higher activation than men, F(1, 113) = 7.61, *p* = 0.007, ηp^2^ = 0.063 (women: M = 2.81; men: M = 2.50). Distributions for both outcomes across the 2 × 2 groups are shown in Figure 3a,b.

## 4. Discussion

The results of this study provide important insights into the use of non-immersive virtual reality as a tool for eliciting complex emotions such as disgust and anxiety. The significant emotional activation observed among participants, reflected by pre–post increases in state anxiety and disgust sensitivity, suggests that this non-immersive virtual scenario can elicit clinically relevant affective responses in a non-clinical sample. Nevertheless, because the study did not include a neutral VR condition or a non-VR control, these findings should be interpreted as preliminary and do not support causal claims regarding the effectiveness of non-immersive VR. Alternative explanations for pre–post changes include repeated measurement effects, expectancy/demand characteristics, and time/order effects (see also Alghamdi et al., 2017; Wan et al., 2024).

The significant increase in state anxiety and disgust sensitivity scores after exposure to the dirty public bathroom scenario highlights the ability of non-immersive VR to evoke intense emotional responses. This is particularly relevant for the potential use of such tools in therapeutic contexts, where the elicitation of specific emotions can be useful for exploring and managing them in a controlled environment. Non-immersive VR could represent a less invasive alternative to immersive VR while still maintaining the ability to engage users and activate emotional responses (Ventura et al., 2019; Rosa et al., 2020; Lima et al., 2024; Mancuso et al., 2024). This is particularly useful for individuals who may not tolerate the use of immersive headsets, such as the elderly or individuals with disabilities.

From a contribution standpoint, the present findings should not be interpreted as a generic confirmation that “VR elicits negative emotions”, as contamination-related disgust/anxiety activation has been previously documented in virtual exposure paradigms (Inozu et al., 2020; Ferraioli et al., 2024). Instead, our results provide evidence that a remote, non-immersive, screen-based implementation, which may be more accessible and scalable than immersive systems, can still produce robust emotional activation within an ecologically plausible contamination scenario. This added value is clinically and practically relevant because immersive technologies can present barriers in terms of accessibility and tolerability (e.g., usability constraints and cybersickness), which may limit deployment in certain populations or settings (Creed et al., 2024; Dilanchian et al., 2021). By empirically testing this specific non-immersive format and examining moderators and vulnerability-linked associations, our study contributes to defining when and for whom screen-based paradigms may be informative and usable, rather than assuming equivalence with immersive VR outcomes (Garay-Sanchez et al., 2021; Gómez Bergin & Craven, 2023).

The observed associations between disgust sensitivity, obsessive–compulsive tendencies, and anxiety-related measures are consistent with prior literature linking these constructs. However, given the correlational nature of the analyses and the non-clinical sample, these relationships should be interpreted as descriptive covariation rather than evidence of underlying mechanisms or clinical processes (Di Natale et al., 2024; Ferraioli et al., 2024). Nevertheless, the correlational results should be interpreted with caution. Many observed associations were small, and statistical significance alone does not establish practical or clinical importance. Correlations cannot identify underlying mechanisms and do not support causal conclusions. We therefore interpret these findings as descriptive evidence of covariation among self-report measures in this sample and context, and we avoid extrapolating them to clinical mechanisms or treatment implications. Emphasizing effect sizes and careful substantive reasoning is consistent with current methodological recommendations for psychological research (Wasserstein & Lazar, 2016; Cumming, 2014; Funder & Ozer, 2019).

The significant differences observed between males and females indicate a greater sensitivity among women to disgust and anxiety stimuli. This is consistent with existing literature, which suggests that women tend to be more sensitive to negative emotional stimuli, especially those related to the risk of contamination (Ding et al., 2021; Üzümcü et al., 2023). Previous experience with first-person videogames seems to attenuate the emotional response to the virtual scenario, suggesting that familiarity with virtual environments may desensitize users to such stimuli. This could imply that the effectiveness of VR as a therapeutic tool may vary depending on the user’s prior experience with the technology. Although DSR scores differed from pre to post exposure, this should not be interpreted as evidence of a stable change in a trait-like disposition. Rather, the post-exposure shift is more plausibly understood as a short-term change in self-report (e.g., priming/salience effects or changes in the accessibility of contamination-related appraisals) following contact with highly salient contamination cues (Olatunji et al., 2025). This interpretation is consistent with evidence that disgust-related dispositions can change in the context of learning/exposure-based interventions, where disgust proneness decreases across treatment (Taboas et al., 2015; Leutgeb et al., 2012).

The present findings should be interpreted in light of the characteristics of the sample and data-collection period. Participants were non-clinical university students, and therefore the observed associations and pre–post changes cannot be assumed to reflect patterns in clinical populations. In addition, the study was conducted during the COVID-19 pandemic, a context in which disgust and contamination-related concerns have been reported to increase at the population level, potentially amplifying responses to contamination cues (Stevenson et al., 2021). Consequently, our results should be regarded as preliminary evidence of affective elicitation in a non-clinical sample within a specific historical period, rather than as direct support for clinical treatment effects.

### 4.1. Potential Applications

The present findings suggest that a remote, screen-based non-immersive contamination scenario can elicit measurable disgust- and anxiety-related responses in a non-clinical sample. While this may inform the development of accessible tools for emotion elicitation and assessment, any clinical, therapeutic, or educational applications remain speculative at this stage. Establishing applied utility will require controlled comparative studies (e.g., neutral VR and non-VR controls), repeated-session protocols, and testing in clinical samples before implications for intervention or training can be drawn.

### 4.2. Study Limitations

Although the results are promising, the study presents some limitations that need to be considered. Conducted on a sample of university students, the study does not represent a clinical population, so the results may not be generalizable to populations with established psychopathological disorders. A major limitation is the single-group pre-post design without any control or comparison condition. Consequently, it is not possible to draw causal inferences or to attribute the observed changes uniquely to the virtual scenario. Future studies should employ controlled designs, including at minimum (1) a neutral/low-emotion VR condition and/or (2) a non-VR control condition, and ideally randomization/counterbalancing and follow-up measurements to evaluate persistence of effects and to rule out alternative explanations (e.g., repeated testing, expectancy/demand effects, and time/order effects).

Future studies should include clinical samples to explore the applicability of the findings in therapeutic contexts. The use of a single virtual scenario (a dirty public bathroom) may also limit the generalizability of the results. Exploring the effectiveness of non-immersive VR across a variety of scenarios that evoke different emotions could determine whether the observed results are specific to disgust or can be generalized to other emotions. A key limitation concerns external validity. The sample comprised exclusively non-clinical university students, and data were collected during the COVID-19 pandemic. This context may have heightened contamination-related vigilance and disgust responsivity, potentially amplifying emotional reactions to the “dirty bathroom” scenario (Stevenson et al., 2021). Accordingly, the present findings cannot be generalized to clinical populations, and they may not readily extend to post-pandemic conditions. Reviews conducted during the pandemic point to heterogeneous changes in contamination/washing symptoms and obsessive-compulsive symptomatology across individuals and samples, reinforcing the need for caution when interpreting results obtained in this period (Grant et al., 2022; Guzick et al., 2021).

The Activation and Engagement Index (AEI) was developed specifically for this study and should therefore be considered an exploratory indicator of subjective engagement and activation during the virtual experience. Although internal consistency was acceptable and the EFA suggested a largely unidimensional pattern, the AEI has not yet been validated through confirmatory factor analysis in an independent sample or through convergent/discriminant and test–retest reliability assessments. Therefore, AEI-related findings should be interpreted cautiously and not as definitive evidence regarding engagement/activation mechanisms (Boateng et al., 2018; Fabrigar et al., 1999).

Another limitation is that emotional activation was assessed exclusively through self-report questionnaires. While self-report provides direct access to subjective experience, it may be influenced by demand characteristics, introspective limits, and response styles, and it does not capture fine-grained temporal dynamics of affective responses during the virtual experience. Consequently, the present findings should be interpreted as evidence of self-reported emotional activation rather than as a comprehensive, objective characterization of emotional responding. Because immedesimation was operationalized using a brief instruction and a single-item discomfort rating, it should be considered an exploratory indicator of situational self-identification rather than a comprehensive measure of embodiment or presence.

The sensitivity power analysis further indicated that, with the present sample size, the study was able to detect effects of approximately dz = 0.26 in paired pre–post comparisons, r = 0.25 in correlational analyses, and f^2^ = 0.12 in the multivariable regression models. While the observed pre–post effects on state anxiety and disgust sensitivity exceeded these thresholds, smaller associations may have remained undetected. Accordingly, null findings and weak effects should be interpreted cautiously and warrant replication in larger samples.

Finally, because the study tested a relatively large set of bivariate correlations, the results should be considered exploratory and hypothesis-generating, with an increased risk of Type I error when many tests are conducted. Replication in independent samples and confirmatory analyses are needed before drawing stronger substantive conclusions (Wasserstein & Lazar, 2016; Cumming, 2014).

### 4.3. Future Directions

Future research should examine this paradigm in clinical samples and adopt controlled, comparative designs to clarify whether screen-based non-immersive exposure yields effects beyond non-specific influences (e.g., expectancy or repeated testing), while also assessing feasibility, acceptability, and symptom-relevant outcomes. Replication in post-pandemic contexts is warranted to determine whether the magnitude and pattern of affective responses observed here persist when contamination threat is less salient at the societal level (Stevenson et al., 2021).

Future studies should also complement self-report with objective physiological measures, ideally adopting a multimethod framework (e.g., EEG and other psychophysiological indices) to capture the temporal unfolding of affect and to strengthen inferences about emotion elicitation. Recent EEG-based emotion-recognition approaches indicate that spatio-temporal and spatial–frequency neural representations can discriminate subtle emotional states with high performance (Hu et al., 2026; Li et al., 2026).

## 5. Conclusions

The findings provide preliminary support for the feasibility of screen-based non-immersive VR scenarios to elicit measurable disgust- and anxiety-related responses in a non-clinical sample. Given the single-group pre–post design without a control condition, the results should be interpreted as associational and do not establish the clinical effectiveness of non-immersive VR. Future research using controlled and comparative designs will be necessary to determine whether and under what conditions non-immersive VR produces effects beyond non-specific factors and whether such effects translate to clinical populations and repeated-session exposure.

By integrating non-immersive VR into treatment plans, therapists and clinicians can offer innovative, accessible, and effective interventions that address a wide range of psychological issues. As technology continues to evolve, further research and development will likely expand the applications of non-immersive VR, making it a valuable tool in the future of mental health care. Non-immersive VR is generally more accessible and cost-effective compared to immersive VR setups. This makes it a practical option for a wide range of clinical settings, including those with limited resources. The flexibility of non-immersive VR allows for easy customization of scenarios to meet the specific needs of different patients. Therapists can tailor the experience to target specific triggers or behaviors, enhancing the effectiveness of the intervention. Some patients may be more willing to engage with non-immersive VR due to its less invasive nature. This could be particularly beneficial for patients who are apprehensive about technology or those who experience discomfort with immersive VR equipment.

These results support the feasibility of using screen-based non-immersive scenarios to elicit contamination-relevant affect in a non-clinical sample. However, clinical utility should not be inferred from the present design and sample. Future controlled studies in clinical populations, using appropriate comparison conditions and repeated-session protocols, are needed to determine whether such paradigms yield incremental benefits over non-specific factors and whether they can be meaningfully integrated into applied settings.

## Figures and Tables

**Table 1:** Bivariate correlations.

	GBD	ID	DTBD	DSD	EMAS-S	EMAS-T	FCS	OCI	DSR
General Bathroom Discomfort (GBD)	1	<0.001	<0.001	<0.001	0.726	0.063	0.150	0.002	0.086
Immedesimation Discomfort (ID)	0.417	1	<0.001	0.009	0.064	0.031	0.127	0.037	0.007
Dirty Toilet Bowl Discomfort (DTBD)	0.431	0.399	1	<0.001	0.922	0.114	0.037	0.007	<0.001
Dirty Sink Discomfort (DSD)	0.433	0.236	0.692	1	0.375	0.241	0.184	0.022	<0.001
EMAS-S	0.032	0.168	−0.009	−0.081	1	<0.001	0.003	0.181	0.034
EMAS-T	0.169	0.195	0.144	0.107	0.295	1	<0.001	0.023	0.007
Fear of COVID-19 Scale (FCS)	0.131	0.139	0.189	0.121	0.267	0.452	1	<0.001	<0.001
Obsessive-Compulsive Inventory (OCI-R)	0.282	0.189	0.243	0.207	0.122	0.206	0.424	1	0.002
Disgust Scale Revised (DSR)	0.156	0.242	0.364	0.317	0.192	0.245	0.331	0.277	1

Note. Lower diagonal = Pearson’s r; upper diagonal = two-tailed *p*-values; N = 122.

## Data Availability

The original contributions presented in this study are included in the Supplementary Material. Further inquiries can be directed to the corresponding author.

## Supplementary Materials

The following supporting information can be downloaded at: https://www.mdpi.com/article/10.3390/bs16030445/s1.
